# Right ventricular strain imaging using 3D SPAMM combined with optical flow tracking

**DOI:** 10.1186/1532-429X-14-S1-W54

**Published:** 2012-02-01

**Authors:** Hazel D Rovno, Chun Xu, James J Pilla, Lawrence Dougherty, Jeremy R McGarvey, Robert C Gorman, Joseph H Gorman, Kevin Koomalsingh, Harold Litt

**Affiliations:** 1 Radiology, University of Pennsylvania, Warren, NJ, USA; 2 Surgery, University of Pennsylvania, Philadelphia, PA, USA

## Summary

A SPAMM sequence and optical flow tracking method that permits evaluation of strain in all 3 dimensions simultaneously in a single acquisition was successfully applied to the right ventricle.

## Background

Presently there is no method of evaluating right ventricle function that simultaneously accounts for its complex geometry and motion. RV strain imaging is promising for evaluating regional dysfunction but to date it has not been possible to assess all 3 dimensions in a single acquisition. A novel variant of SPAMM tagging combined with optical flow tracking is available at our institution that can evaluate strain and torsion simultaneously in all 3 dimensions. We have applied this method to the right ventricle.

## Methods

Data was analyzed, with attention to the right ventricle, from data acquired during a study of left ventricular remodeling post acute infarction. Imaging was obtained using respiratory triggering, with animals ventilated under general anesthesia, and triggering from invasive monitoring of left-sided pressures. 3-D tagging was performed at early (less than one week) and later (4-6 week) postoperative time points. Typical parameters for the 3-D tagging sequence at high resolution are: slice thickness 1mmx0.5mmx2mm interpolated to 0.5x0.5x2mm; 4mm tag spacing; coverage of about half of the heart, at mid-ventricle level; 6 nex; 30 minute acquisition time with respiratory gating. 5 or 6 phases were acquired during average systole. Typical parameters for the sheep 3-D tagging sequence at low resolution are: slice thickness 2mmx1mmx2mm interpolated to 1x1x2 ; whole heart coverage; 6mm tag spacing; 4 nex (4 signal acquisitions); 45 minute acquisition time with respiratory gating. Tag tracking was performed using an optical flow method and principal strain was calculated from the displacement using a custom program.

## Results

It is consistently possible, with this sequence, to obtain regional principal strain of the right ventricle over systole. Vectors obtained and strain values obtained were compared to expectations of the porcine right ventricle (Cho AmJP2009). Septal strains had a mean of approximately 20%. RV free wall strains were high for a 5-week postop animal at around 80-110%; for a different animal, 7 days postop, RV free wall strain was around 27-60%. These are within range of expected values.

## Conclusions

It is possible to measure right ventricular strain in 3 dimensions with a single acquisition, using a variant of SPAMM tagging. The technique permits regional analysis of right ventricular strain. Additionally, it holds promise for analysis of time-resolved regional strain in the right ventricle, though that was not consistently possible in the images analyzed to date.

Further refinements are expected to include imaging in the long axis as well as short axis for better resolution in the direction of expected greatest strain, and adjustment of the postprocessing software to permit subanalysis of strain in radial and long axis dimensions. Having now shown that the sequence is applicable to the right ventricle, we anticipate adjusting the sequence to permit clinical human applications.

## Funding

None.

**Figure 1:**
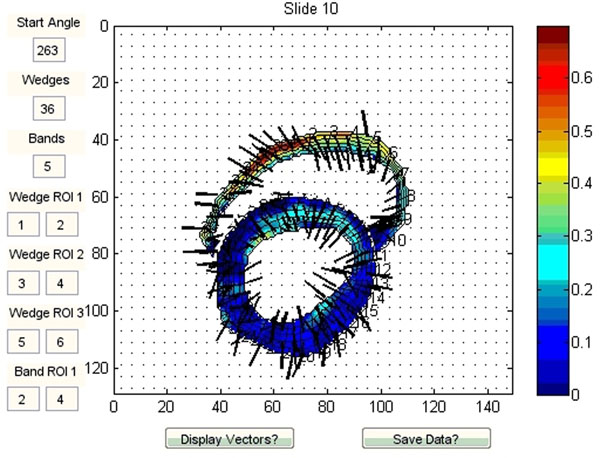
Short axis mid-ventricular slice from a low-resolution acquisition. Note - because of current limitations in the software, it is not possible to obtain this contour in a single acquisition. This is a composite of 1) an RV-LV free wall (no septum) acquisition, and 2) a superimposed LV image with identical parameters. Vectors are superimposed, showing direction of maximum principal strain.

**Figure 2:**
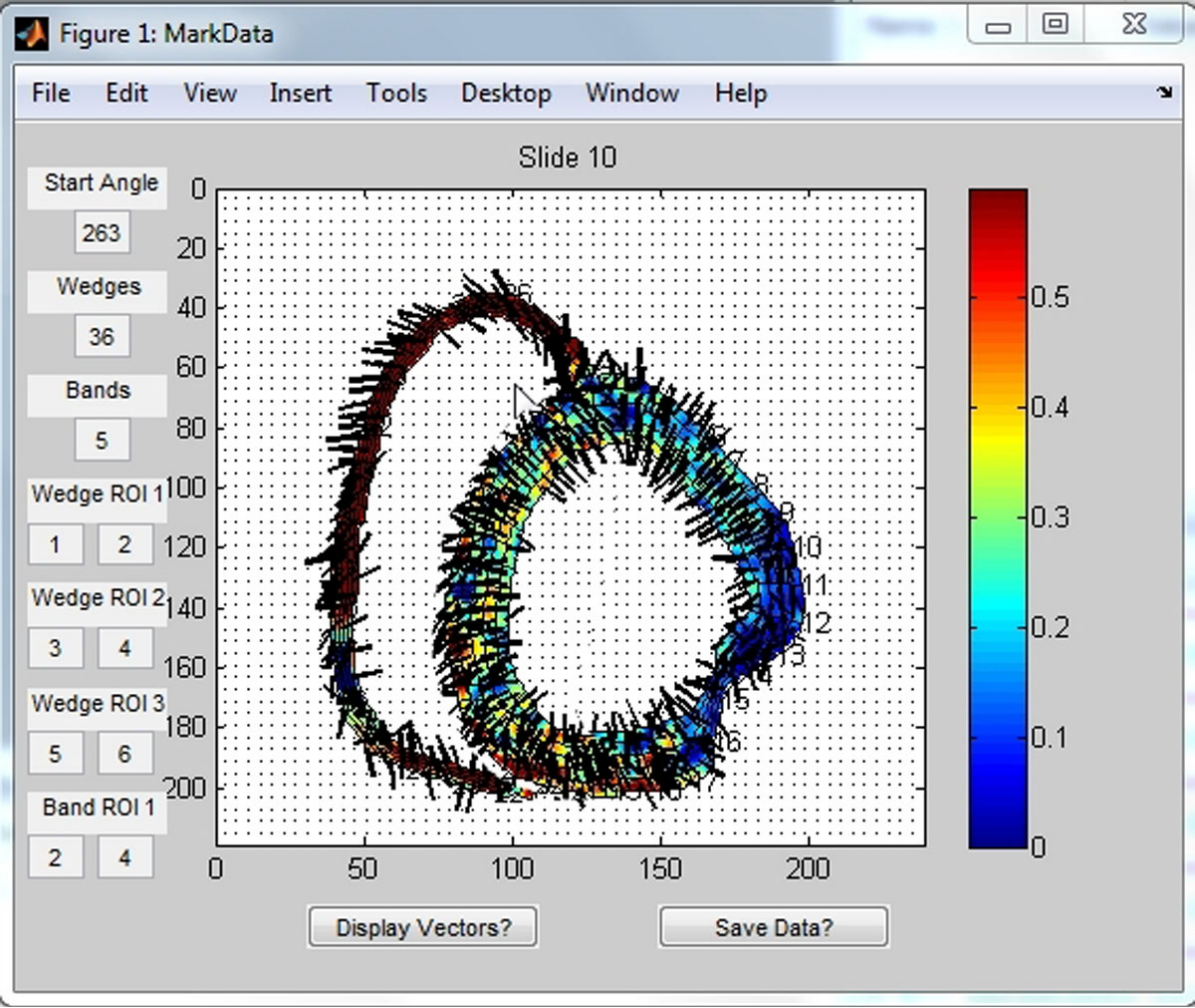
Short axis mid-ventricular slice, a high-resolution acquistion, different animal. Note - because of current limitations in the software, it is not possible to obtain this contour in a single acquisition. This is a composite of 1) an RV-LV free wall (no septum) acquisition, and 2) a superimposed LV image with identical parameters. The strain vectors are superimposed. Note that the predominant directions of strain correspond to that expected.

